# Isolation and Characterization of Phosphorus Solubilizing Bacteria With Multiple Phosphorus Sources Utilizing Capability and Their Potential for Lead Immobilization in Soil

**DOI:** 10.3389/fmicb.2020.00752

**Published:** 2020-04-23

**Authors:** Wenjie Wan, Yin Qin, Huiqin Wu, Wenlong Zuo, Huangmei He, Jiadan Tan, Yi Wang, Donglan He

**Affiliations:** ^1^College of Life Science, South-Central University for Nationalities, Wuhan, China; ^2^State Key Laboratory of Agricultural Microbiology, Huazhong Agricultural University, Wuhan, China

**Keywords:** *Acinetobacter pittii* gp-1, multiple phosphorus source utilizing capacity, Pb immobilization, P-cycling-related gene, phosphorus solubilizing bacteria, *ppk* and *pqq* genes

## Abstract

Phosphorus solubilizing bacteria (PSB) can promote the level of plant-absorbable phosphorus (P) in agro-ecosystems. However, little attention has been paid to PSB harboring abilities in utilizing multiple phosphorus sources and their potentials for heavy metal immobilization. In this study, we applied the strategy of stepwise acclimation by using Ca_3_(PO_4_)_2_, phytate, FePO_4_, and AlPO_4_ as sole P source. We gained 18 PSB possessing abilities of multiple P sources utilization, and these bacteria belonged to eight genera (*Acinetobacter*, *Pseudomonas*, *Massilia, Bacillus*, *Arthrobacter*, *Stenotrophomonas*, *Ochrobactrum*, and *Cupriavidus*), and clustered to two apparent parts: Gram-positive bacteria and Gram-negative bacteria. The isolate of *Acinetobacter pittii* gp-1 presented good performance for utilizing Ca_3_(PO_4_)_2_, FePO_4_, AlPO_4_, and phytate, with corresponding P solubilizing levels were 250.77, 46.10, 81.99, and 7.91 mg/L PO_4_^3–^-P, respectively. The PSB *A. pittii* gp-1 exhibited good performance for solubilizing tricalcium phosphate in soil incubation experiments, with the highest values of water soluble P and available P were 0.80 and 1.64 mg/L, respectively. Additionally, the addition of *A. pittii* gp-1 could promote the immobilization of lead (Pb), and the highest Pb immobilization efficiency reached 23%. Simultaneously, we found the increases in abundances of both alkaline phosphatase gene (*phoD*) and β-propeller phytase gene (*bpp*) in strain gp-1 added soils. Besides, we observed the expression up-regulation of both pyrroloquinoline quinone gene (*pqq*) and polyphosphate kinases gene *(ppk*), with the highest relative expression levels of 18.18 and 5.23, respectively. We also found the polyphosphate particles using granule staining. To our knowledge, our findings first suggest that the solubilizing of tricalcium phosphate by phosphorus solubilizing bacterium belonging to *Acinetobacter* is coupled with the synthesis of polyphosphate. Taken together, *A. pittii* gp-1 could be a good candidate in improving soil fertility and quality.

## Introduction

Phosphorus (P) is the second limiting nutrient required for plant growth and development involved in important metabolic pathways like nutrient uptake, biological oxidation, and energy metabolism (Nesme et al., 2018). The total P in soil accounts roughly for 0.04–0.1% (w/w), only a very tiny proportion of P (soluble H_2_PO_4_^–^ or HPO_4_^2–^) can directly be assimilated by plants (Chen et al., 2008), as the large portion of P in soils exists in inorganic insoluble form [e.g., Ca_3_(PO_4_)_2_] and organic insoluble/soluble form (e.g., phytate and nucleic acid) (Liu et al., 2015; Neal et al., 2017). The input of P to soil is mainly via fertilization, and both abiotic substance (mainly inorganic P mineral) and organic P compounds (e.g., animal, plant, and microbes residues and wastes) are widely used in agricultural ecosystems (Lim et al., 2007; Jorquera et al., 2013; Fraser et al., 2015), and these extraneously added P including inorganic P (IP) and organic P (OP) get converted into salts and become insoluble by bounding to Ca, Al, Mg, Mn, and Fe (Liu et al., 2015; Reddy et al., 2015). Insoluble IP especially Ca_3_(PO_4_)_2_, AlPO_4_, and FePO_4_, and insoluble/soluble OP especially phytate taking up 80% of soil OP need phosphorus-solubilizing microbes (PSM) to transform into orthophosphate which can be absorbed by plants and microbes (Lim et al., 2007; Liu et al., 2014; Fraser et al., 2015). Previous literatures have reported that insoluble IP can be dissolved by low molecular weight organic acids (e.g., citric and gluconic acids) produced and released by both phosphorus solubilizing bacteria (PSB) and fungi (Sashidhar and Podile, 2009; Ogbo, 2010; Patel et al., 2011), and OP can be digested by extracellular enzymes (e.g., phosphatase and phytase) mainly synthesized and secreted by microbes (Tan et al., 2016; Neal et al., 2017). Previous literatures have reported that repeated utilization of fertilizers exacerbates soil quality and lessens phosphorus availability (Bhattacharyya et al., 2015; Liu et al., 2018). To achieve the aim of sustainable agriculture, the application of phosphorus solubilizing microbes with multiple P sources utilizing abilities provides a new approach to improve soil quality.

Phosphorus solubilizing microbes especially PSB are widely distributed in soils, freshwater, seawater, and sediments (Liu et al., 2014, 2017; Zhang et al., 2015), and responsible for the cycling of insoluble P to soluble PO_4_^3–^ ion. Numerous researches have concentrated on the screening of highly efficient PSB, and most PSB are Gram-negative bacteria and belong to *Pseudomonas* (Misra et al., 2012; Oteino et al., 2015), *Acinetobacter* (Liu et al., 2014, 2015), *Pantoea* and *Enterobacter* (Park et al., 2011; Chen and Liu, 2019), and some PSB are Gram-positive bacteria belonging to *Bacillus* (Hanif et al., 2015; Wang et al., 2017). PSB have showed good performance for plant growth promotion (Patel et al., 2011; Oteino et al., 2015; Wen et al., 2019) and heavy metal immobilization (Park et al., 2011; Yuan et al., 2017; Chen et al., 2019). However, few studies have been conducted to explore the potentials of PSB for dissolving multiple P sources, and the effect of PSB addition on both soil inorganic and organic P-cycling-related gene abundance remains unknown.

Phospholipids and phytate are major organic P pool in soils, which can be hydrolyzed by phosphatase and phytase, respectively (Maougal et al., 2014; Neal et al., 2017; Irshad and Yergeau, 2018). Previous literatures have reported that alkaline phosphatase is exclusively originated from soil microorganisms, and acid phosphatase is mainly produced and secreted by plants (Krämer and Green, 2000; Fraser et al., 2015). Three prokaryotic genes, *phoX*, *phoA*, and *phoD*, are responsible for encoding alkaline phosphatase (Huang et al., 2009); three genes of *bpp* encoding β-propeller phytase, *ptp* encoding protein tyrosine phosphatase, and *hap* encoding histidine acid phosphatase are found in prokaryote (Lim et al., 2007; Neal et al., 2017). However, in terrestrial ecosystems, *phoD*-harboring bacteria are more widely distributed than *phoX*-, *phoA*-harboring bacteria (Neal et al., 2017; Hu et al., 2018), and *bpp*-harboring bacteria are more frequently found than *ptp*-, *hap*-harboring bacteria (Lim et al., 2007; Neal et al., 2017). In addition, the hydrolysis of inorganic insoluble P requires the participation of small molecular organic acid (e.g., gluconic acid, lactic acid, and citric acid), which can be released by *gcd*-harboring bacteria (Vyas and Gulati, 2009; Hanif et al., 2015; Rasul et al., 2019). Therefore, the *phoD*, *bpp*, and *gcd* genes could be good biomarkers in evaluating soil inorganic and organic P transformation.

In Gram-negative bacteria, gluconic acid is produced by the direct oxidation of glucose mediated by a membrane-bound glucose dehydrogenase (GDH), which needs pyrroloquinoline quinone (PQQ) as a cofactor in dehydrogenase reactions catalyzed by the so called quinoproteins (Farhat et al., 2013; Wagh et al., 2014). Quinoproteins exist in many bacteria, like alcohol dehydrogenase from *Pseudomonas aeruginosa*, GDH from *Acinetobacter calcoace*, methylamine dehydrogenase from *Thiobacillus* species (Goosen et al., 1992). The PQQ biosynthesis pathway in bacteria involves several genes presenting in a cluster, whose composition in terms of gene number and their organization exhibits considerable distinction in different species (Choi et al., 2008). Polyphosphate kinases (PPK) encoded by *ppk* gene are responses for the synthesis of polyphosphate considered as a key high-energy compound (Ishige and Noguchi, 2000). Extensive effort has been implemented to determine the *pqq* gene expression level in the process of inorganic phosphorus solubilization, however, little literature has reported the expression level of PPK gene in the process of inorganic phosphorus solubilization. To enrich the knowledge of phosphorus metabolism during inorganic P solubilizing, the gene expression level of *pqq* and *ppk* should be determined.

In this study, we aimed to (i) screen PSB possessing multiple P source utilization capacity, (ii) explore the potential of PSB for lead immobilization, and (iii) determine abundances of P-cycling-related genes in both cells and soils.

## Materials and Methods

### Acclimation, Isolation, and Identification of Phosphorus Solubilizing Bacteria

The NBRIP medium containing 10 g/L glucose, 0.25 g/L MgSO_4_⋅7H_2_O, 5 g/L Ca_3_(PO_4_)_2_/sodium phytate/FePO_4_/AlPO_4_, 5 g/L MgCl_2_⋅7H_2_O, 0.2 g/L KCl, 0.1 g/L (NH_4_)_2_SO_4_, 2 mL/L trace element solution was applied to acclimate and isolate PSB (Misra et al., 2012). Trace element solution contained (g/L) EDTA, 10; MnSO_4_⋅H_2_O, 2.2; FeSO_4_⋅7H_2_O, 1.0; CuSO_4_⋅5H_2_O, 0.5; CoCl_2_⋅6H_2_O, 0.3; Na_2_MoO_4_⋅2H_2_O, 0.2; and CaCl_2_, 0.1. The initial pH of all mediums was adjusted to 7.0 using 1 mol/L NaOH and 1 mol/L HCl solution.

Five gram of bulk soil collected from Laiyang Experimental Station in Shandong Province, China, were added to 50 mL of sterile water and shaken at 180 rpm for 30 min, and the mixture was kept stand for 10 min. In the first round of acclimation, 10 mL of soil suspension were inoculated to 100 mL of liquid NBRIP medium containing Ca_3_(PO4)_2_, and incubated at 30°C with shaking of 180 rpm for 7 days. The microbial suspension was collected and centrifuged at 5000 rpm for 10 min, and then microbial suspension was resuspended with 10 mL of sterile water after precipitation was washed twice. Taking the first round acclimation procedure, the microbial suspension was inoculated to liquid NBRIP containing sodium phytate, FePO_4_ or AlPO_4_, respectively, and incubated at the same condition for another 7 days. The acclimated microbial suspension was designated as A1 (NBRIP containing Ca_3_(PO_4_)_2_), A2 (NBRIP containing sodium phytate), A3 (NBRIP containing FePO_4_), and A4 (NBRIP containing AlPO_4_), respectively. Additionally, part of the microbial suspension of A1, A2, A3, and A4 were centrifuged at 5000 rpm, and the microbial precipitations were stored at −80°C for subsequent DNA extraction.

Taking stepwise dilution strategy, 0.1 mL of 10^–5^ and 10^–8^ diluent of microbial suspension A4 was evenly spread on NBRIP containing Ca_3_(PO_4_)_2_ agar medium and incubated at 30°C for 5 days. The screening strategy for isolation of PSB was by picking colony with clear halo zone. Single colonies were sub-cultured by picking and streaking five times to isolate pure colonies. A total of 18 strains (Ap-1, Ap-2, Ap-3, Ap-4, Ap-6, Ap-8, Ap-9, Ap-10, gp-1, gp-2, gp-3, gp-4, gp-6, gp-7, gp-8, gp-11, gp-12, and gp-15) were gained and identified by simple 16S rRNA gene sequencing in Wuhan Qingke innovation Biotechnology Co., Ltd.

### DNA Extraction, High Throughput Sequencing, and Sequence Processing

The total DNA of microbial precipitations and soil were extracted using a DNA extraction kit (MoBio, Carlsbad, CA, United States) according to the manufacture’s instruction. The DNA concentrations were measured using a NanoDrop 2000 Spectrophotometer (Thermo Fisher Scientific, Waltham, MA, United States). All DNA samples were stored at −80°C.

To determine the bacterial community composition of microbial precipitations (A1, A2, A3, and A4), the V3–V4 region of bacterial 16S rRNA gene was amplified using the primers 338F (5′- ACT CCT ACG GGA GGC AGC A-3′) and 806R (5′- GGA CTA CHV GGG TWT CTA AT-3′) (Mori et al., 2013). The 16S rRNA gene fragments were amplified under the following condition: an initial denaturation at 95°C for 3 min, 30 cycles of 95°C for 40 s, 58°C for 40 s, and 72°C for 50 s, and a final extension at 72°C for 10 min. The triplicate PCR products were pooled and purified by gel electrophoresis, and quantified using a QuantiFluor^TM^ -ST (Promega, United States). Sequencing was carried out on an Illumina Miseq platform at Majorbio Bio-Pharm Technology Co., Ltd., Shanghai, China. The Miseq raw reads were deposited in the National Center for Biotechnology Information (NCBI^1^) Short Read Archive (SRA) database under accession numbers SRR8731888–SRR8731891.

The raw reads were purified following the pathway of Mothur (Schloss et al., 2009). To minimize the effects of random-sequencing errors, we removed (i) sequences that did not exactly match barcodes and primers; (ii) sequences that contained ambiguous bases call; (iii) sequences with an average quality score <20; and (iv) sequences with maximum homopolymers <10 bp. The purified sequences were clustered into operational taxonomic unit (OTU) at 97% identity against the SILVA v128 reference. The community diversity in different acclimation periods was compared using α-diversity indices including Shannon index, ACE, and Chao1.

### Determination of Phosphorus Solubilizing Ability

The 18 strains were separately inoculated to LB medium and incubated at 30°C with shaking of 180 rpm for 16 h. Strains were collected by centrifuging and washed twice with sterile water, and subsequently resuspended. The optical density (600 nm) was adjusted to 1.0 by dilution of the strain pellets with sterile water. These mixtures were designated as seed cultures (OD = 1.0), and separately inoculated to NBRIP solid and liquid medium containing Ca_3_(PO_4_)_2_, sodium phytate, FePO_4_, or AlPO_4_, and incubated at 30°C for 5 days. After incubation, 1 mL of bacterial suspension was collected to determine soluble P concentration, and the diameter of the halo (HD) and the diameter of colony (CD) on solid medium plates were measured (Liu et al., 2015). The PO_4_^3–^-P content in bacterial suspension was determined using molybdenum blue method (Waterlot, 2018).

To determine P solubilizing capacities of the 18 isolated PSB, soils used in incubation experiment were collected from an uncultivated field in Wuhan, middle of China (30°28′N, 114°21′E). The original pH, TC, TN, water soluble P (WSP), Olsen P, total P, and total lead were 6.92, 0.52%, 0.68%, 0.09 mg/g, 0.22 mg/g, 0.89 mg/g, and 0.57 mg/g, respectively. Three groups with three replicates were designed: 100 g soil + 10 mL seed cultures (S + B group), 95 g soil + 10 mL seed cultures + 5 g Ca_3_(PO4)_2_ (S + B + T group), 95 g soil + 10 mL seed cultures + 5 g Ca_3_(PO4)_2_ + 10 mL NBRIP liquid medium without P sources (S + B + T + N group). Sterile water was added to adjust soil moisture to 80%, and these experimental groups were incubated at room temperature for 30 days. After incubation, soils were collected to determine physicochemical properties, including the content of WSP and available P (AP). The WAP and AP were extracted by ultrapure water and 0.5 mol L^–1^ NaHCO_3_ (pH 8.5), respectively, at a ratio of 1:20 of soil to solution, and the extracted solutions were then filtered through Whatman Grade No. 42 Quantitative Filter Paper (Olsen et al., 1954), and then were measured using molybdenum blue method.

### Determination of Lead Immobilization by PSB gp-1

To evaluate the effect of PSB gp-1 inoculation on heavy metal immobilization, the soils containing weight ratio of 1, 2, 3, 4, 5, and 10% Ca_3_(PO4)_2_ were spiked with Pb(NO_3_)_2_ at a concentration of 500 mg Pb/kg soil and incubated at room temperature for 30 days. No strain gp-1 addition but with Ca_3_(PO4)_2_ addition, and without both strain gp-1 and Ca_3_(PO4)_2_ addition were used as blank control. Every 5 days, sterile water was added to maintain the moisture value of 80%. After incubation, soils were collected and dried at 60°C, then ground and sieved through a 0.150 mm mesh for available heavy metal evaluation. Heavy metal contaminated soils were extracted with acetic acid (HAc) solution (0.11 mol/L) with a soil to solution ratio of 1:40 and shaken at 180 rpm for 16 h (Yuan et al., 2017). The concentrations of Pb(II) in extraction solutions were measured at an AA240FS atomic-absorption spectrophotometer (Varian Company, United States). Additionally, the content of WSP and AP in heavy metal contaminated soils was determined.

### Determination of P-Cycling-Related Gene Abundance

The absolute abundances of P-cycling-related genes in Pb contaminated soil were measured using quantitative PCR (qPCR). Primer sequences for the target genes were applied for qPCR based on previous literatures. Primer bppF (5′–GAC GCA GCC GAY GAY CCN GCN NTN TGG–3′) and primer bppR (5′–CAG GSC GCA NRT CAN CRT TRT T–3′) were employed to amplify *bpp* gene (Huang et al., 2009); primer ALPS-F730 (5′–CAG TGG GAC GAC CAC GAG GT–3′) and primer ALPS-1101 (5′–GAG GCC GAT CGG CAT GTC G–3′) were applied to amplify *phoD* gene (Hu et al., 2018); primer gcdF (5′–CGG CGT CAT CCG GGS NTN YRA YRT–3′) and primer gcdR (5′–GGG CAT GTC CAT GTC CCA NAD RTC RTG–3′) were used to amplify *gcd* gene (Cleton-Jansen et al., 1990). Additionally, primer Eub338 (5′–ACT CCT ACG GGA GGC AGC AG–3′) and primer Eub518 (5′-ATT ACC GCG GCT GG-3′) (Fierer et al., 2005) were selected to amplify 16S rRNA gene. Standard curves were generated using a 10-fold serial dilution of a known amount of recombinant plasmid containing specific gene fragment. Quantitation was performed on three technical replicates with an Applied Biosystems QuantStudio3 Real-time PCR System (Applied Biosystems, Foster City, CA, United States) in a 10 μL reaction system, and was implemented at 95°C for 5 min, followed by 40 cycles of 95°C for 15 s and 55°C for 1 min. The abundances of all genes were expressed as copies per gram of freeze-dried soil. In addition, we employed these primers to amplify *bpp*, *phoD*, and *gcd* from *Acinetobacter pittii* gp-1.

### Identification and Quantitation *ppk* and *pqq* Gene

Genomic DNA- extracted from strain gp-1 was used as template for amplification of the *ppk* and *pqq* genes. According to the gene sequences encoding PPK and PQQ in several related bacteria, two conservative DNA fragments were discovered with the DNAMAN program with default parameters for each gene. Primer F1 (5′–ATG ACA GAA GGT GTT GGC CT–3′) and primer R1 (5′–CTA TAT ATC TGT AAT CGT GTG GAC T–3′) were used to amplify the whole *pqq* gene. While primer F2 (5′–ATG AAT ACA GCG ATT ACA CCA A–3′) and primer R2 (5′–TTA TTT AAT AAT TTC TAA TAG TGC CCT TTG–3′) were employed to amplify the whole *ppk* gene. The acquired *pqq* gene had 1155 bp coding a polypeptide of 384 amino acids (accession number: MG820120). The obtained *ppk* gene had 2079 bp coding a polypeptide of 692 amino acids (accession number: MG820119). The obtained gene sequences were aligned with the closely related sequences extracted by BLAST^2^ from the GenBank database.

In this assay, the strain gp-1 was inoculated to NBRIP with a shaking speed of 150 rpm at 30°C. The cells were collected at the 0, 6, 9, 12, and 24 h for extracting total RNA by using TriZol reagent. The cDNA synthesis was conducted using a CellAmp^TM^ Direct RT-qPCR Kit (Takara Biotech, Beijing, China). To quantitative analysis of gene expression, primer 16S-F (5′–GCG GAG AGA AGT AGC TTG CT–3′) and primer 16S-R (5′–CCG ACT TAG GCT CAT CTA TTA G–3′) were used to amplify 16S rRNA gene; primer ppk-F (5′–CCA TTT ACC TTA CAT GCT CAG CT–3′) and primer ppk-R (5′–GGT CAA TCT GAA CAC CTG CTT–3′) were applied to amplify *ppk* gene; primer pqq-F (5′–GAT GCC TTA GCA GGT TCA AA–3′) and primer pqq-R (5′–CTC AAC TGT ATC TGC ATT AAG TT–3′) were employed to amplify *pqq* gene. The three pairs of primers were designed based on the complete gene sequence data obtained in this assay. The abundance of 16S rRNA gene was used as inner control for copy number estimation. The qPCR was performed using SYBR green PCR Master Mix (Applied Biosystems) and reactions were operated in a real time PCR system (Applied Biosystems 3) in the following steps: denaturation at 94°C for 10 min, followed by 40 cycles of denaturation at 94°C for 15 s, annealing at 60°C for 1 min. Additionally, the concentration of extracellular phosphorus at each time point was also measured. Simultaneously, the granule staining was conducted to investigate the existence of polyphosphate (Poly-P) according to the method described in previous literature (Wan et al., 2017).

### Data Analysis

Significant differences were obtained by the one-way analysis of variance (one-way ANOVA) with means compared using the Tukey test in IBM SPSS 19. A neighbor-joining phylogenetic tree with 1000 bootstrap replicates was built based on 16S rRNA gene sequence using MEGA6 (Tamura et al., 2013). The immobilized Pb was assessed using the equation: Pb immobilization efficiency = [HAc extractable Pb (Soil + Ca_3_(PO_4_)_2_)−HAc extractable Pb (Soil + Ca_3_(PO_4_)_2_ + PSB)] / HAc extractable Pb (Soil + Ca_3_(PO_4_)_2_) × 100%

## Results

### Shifts in Diversity and Composition of Bacterial Community During Acclimation

During the four acclimation periods, the α-diversity indices including OTU (32–62), ACE (36–64), Chao1 (35–68), and Shannon index (1.30–2.23) decreased (Table 1), suggesting the species diversity gradually decreased. The Good’s coverage of all samples was 99.99%, suggesting the amplicon libraries could represent most of the species in the natural habitat.

**TABLE 1:** The bacterial α-diversity indexes in four acclimation period.

Sample	OTU	Chao1	ACE	Shannon	Good’s coverage (%)
A1	62	68	64	2.23	99.99
A2	51	54	57	2.02	99.99
A3	32	35	36	1.30	99.99
A4	32	35	36	1.30	99.99

**FIGURE 1:**
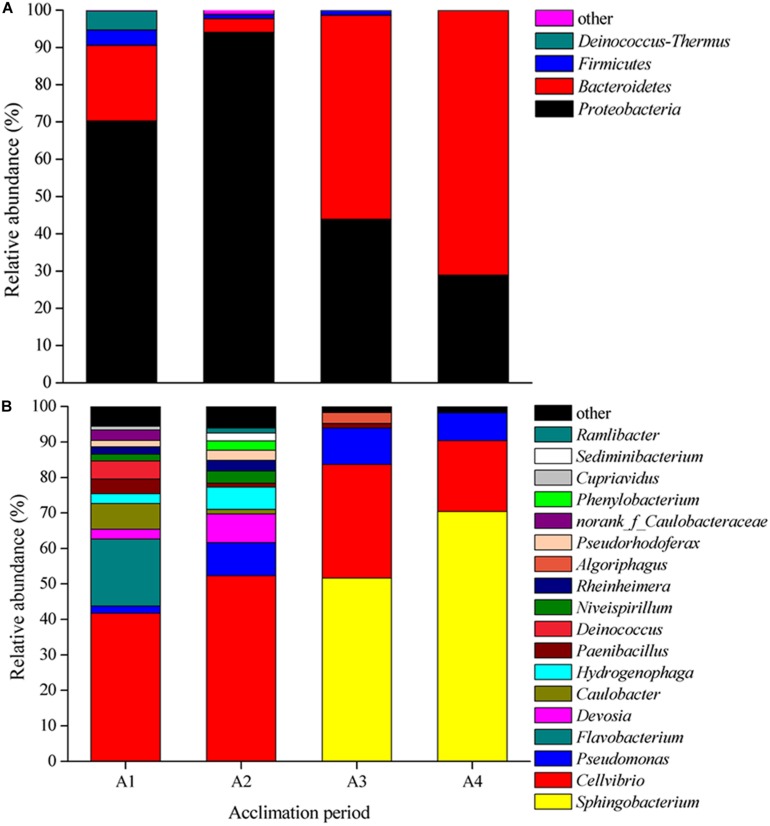
Shifts in bacterial community composition during four acclimation stages. **(A)** Relative abundance of 16S rRNA gene sequences classified to phylum level. **(B)** Relative abundance of 16S rRNA gene sequences classified to genus level.

The composition of bacterial community at both phylum and genus level presented large differences in four acclimation periods (Figure 1). At the phylum level, *Proteobacteria* was the dominant phylum in A1 (70.28%) and A2 (94.02%), and *Bacteroidetes* dominated in A3 (54.74%) and A4 (70.99%) (Figure 1A). while *Firmicutes* and *Deinococcus–Thermus* were the secondary level bacteria. At the genus level, *Cellvibrio* was the first dominant genus in A1 (41.72%) and A2 (52.3%), while *Sphingobacterium* was the supreme dominant genus in A3 (51.64%) and A4 (70.40%) (Figure 1B). The total relative abundances of *Sphingobacterium*, *Cellvibrio*, and *Pseudomonas* in A3 and A4 could reach to 93.88 and 98.2%, respectively. In these periods, *Algoriphagus*, *Caulobacter*, *Caulobacteraceae*, *Cupriavidus*, *Deinococcus*, *Devosia*, *Flavobacterium*, *Hydrogenophaga*, *Niveispirillum*, *Paenibacillus*, *Phenylobacterium*, *Pseudomonas*, *Pseudorhodoferax*, *Ramlibacter, Rheinheimera*, and *Sediminibacterium* exhibited low abundances. There results indicated that species diversity declined and bacterial community composition changed during long-term acclimation.

### Phosphorus Solubilizing Bacteria and Phosphorus Solubilizing Characteristics

**FIGURE 2:**
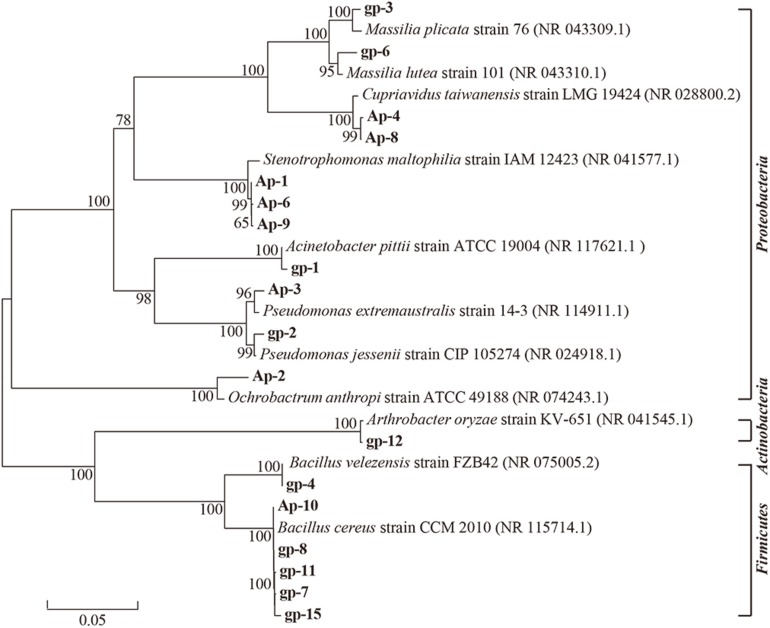
Neighbor-joining phylogenetic tree of 18 phosphorus solubilizing bacteria based on 16S rRNA gene sequences. The numbers at the nodes indicate the levels of bootstrap support based on data for 1000 replicates. The scale bar represents 0.05 sequence divergence.

These 18 PSB were identified as *Acinetobacter* (gp-1), *Pseudomonas* (gp-2 and Ap-3), *Massilia* (gp-3 and gp-6), *Bacillus* (gp-4, gp-7, gp-8, gp-11, gp-15, and Ap-10), *Arthrobacter* (gp-12), *Stenotrophomonas* (AP-1, Ap-6, and Ap-9), *Ochrobactrum* (Ap-2), and *Cupriavidus* (Ap-4 and Ap-8) based on 16S rRNA gene sequence (Table 2). The colony morphology of most strains presented circular, wet texture, and entire edge with the exception of *Bacillus* strain, and most of them exhibited off-yellow and crateriform (Table 2). A phylogenetic tree was built to reflect the phylogenetic distance between these PSB (Figure 2). The 18 strains were clustered into two distinct parts: Gram-negative bacteria and Gram-positive bacteria. Besides, these Gram-negative bacteria belonged to *Proteobacteria*, and Gram-positive bacteria belonged to *Firmicutes* and *Actinobacteria*.

**TABLE 2:** Colony morphologies and identities of the PSB strains.

Strains	Length^a^	Colony M^b^	Most closely related species	Accession number	% identity
gp-1	1428	C, W, E, Cr, W	*Acinetobacter pittii* ATCC 19004 (NR_117621.1)	MK641660	99.23
gp-2	1498	C, W, E, Cr, OY	*Pseudomonas jessenii* CIP 105274 (NR_024918.1)	MK641658	98.86
gp-3	1432	C, W, E, Cr, OY	*Massilia plicata* 76 (NR_043309.1)	MK641663	99.37
gp-4	1511	EI, D, I, R, OY	*Bacillus velezensis* FZB42 (NR_075005.2)	MK641661	99.93
gp-6	1437	C, W, E, Cr, OY	*Massilia lutea* 101 (NR_043310.1)	MK641662	98.73
gp-7	1513	C, D, E, Cr, Y	*Bacillus cereus* CCM 2010 (NR_115714.1)	MK641665	99.93
gp-8	1513	EI, D, I, Cr, OY	*Bacillus cereus* CCM 2010 (NR_115714.1)	MK642886	100
gp-11	1513	EI, D, I, Cr, OY	*Bacillus cereus* CCM 2010 (NR_115714.1)	MK634591	99.93
gp-12	1461	C, W, E, Cr, OY	*Arthrobacter oryzae* KV-651 (NR_041545.1)	MK641666	99.51
gp-15	1512	EI, D, I, Cr, OY	*Bacillus cereus* CCM 2010 (NR_157734.1)	MK634592	99.73
Ap-1	1508	C, W, E, Cr, W	*Stenotrophomonas maltophilia* IAM 12423 (NR_041577.1)	MK641655	98.87
Ap-2	1444	C, W, E, Cr, OY	*Ochrobactrum anthropi* ATCC 49188 (NR_074342.1)	MK641654	98.13
Ap-3	1498	C, W, E, Cr, OY	*Pseudomonas extremaustralis* 14-3 (NR_114911.1)	MK641653	99.33
Ap-4	1493	C, W, E, Cr, OY	*Cupriavidus taiwanensis* LMG 19424 (NR_028800.2)	MK641656	98.99
Ap-6	1508	C, W, E, Cr, W	*Stenotrophomonas maltophilia* IAM 12423 (NR_041577.1)	MK641664	98.80
Ap-8	1495	C, W, E, Cr, OY	*Cupriavidus taiwanensis* LMG 19424 (NR_028800.2)	MK641659	98.99
Ap-9	1508	C, W, E, Cr, W	*Stenotrophomonas maltophilia* IAM 12423 (NR_041577.1)	MK641667	98.80
Ap-10	1513	EI, D, I, Cr, OY	*Bacillus cereus* CCM 2010 (NR_115714.1)	MK641657	100

*^a^Length: 16S rRNA gene sequence length. ^b^Colony M, colony morphology on NBRIP containing Ca_3_(PO_4_)_2_ solid medium. C/EI, circular/ellipse; W/D, wet/dry; E/I, entire/irregular edge; R/Cr, raised/crateriform: Y/OY/W, yellow/off-white/white.*

**FIGURE 3:**
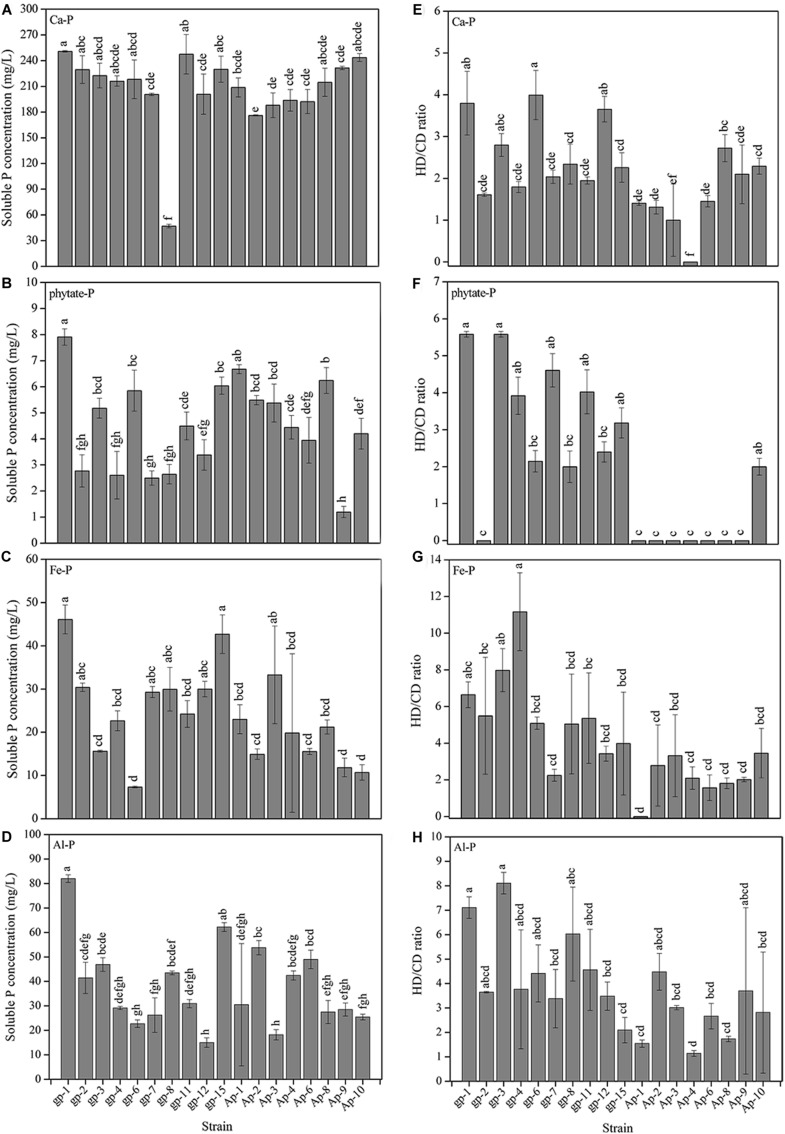
The P solubilizing levels of 18 PSB in liquid NBRIP medium containing Ca_3_(PO_4_)_2_ **(A)**, sodium phytate **(B)**, FePO_4_ **(C)**, and AlPO_4_ **(D)**, and in solid NBRIP medium containing Ca_3_(PO_4_)_2_ **(E)**, sodium phytate **(F)**, FePO_4_ **(G)**, and AlPO_4_ **(H)**. The results are the mean value of three replicates, error bars represent standard error. The lowercase letters above the columns denote significant level (*P* < 0.05).

The 18 PSB presented different abilities to utilize Ca_3_(PO_4_)_2_, phytate, FePO_4_, and AlPO_4_ (Figure 3). Basically, Ca_3_(PO_4_)_2_ was the optimal P source for these 18 PSB in liquid medium (Figure 3A), followed by AlPO4 (Figure 3D), FePO4 (Figure 3C), and phytate (Figure 3B). The P dissolving level of these 18 strains in NBRIP liquid medium separately containing Ca_3_(PO_4_)_2_, phytate, FePO_4_, and AlPO_4_, were 47.08–250.77, 1.19–7.91, 7.35–46.10, and 14.99–81.99 mg/L PO_4_^3–^-P, respectively. Additionally, the P solubilizing level represented by HD/CD ratio of these 18 strains in NBRIP solid medium separately containing Ca_3_(PO_4_)_2_, phytate, FePO_4_, and AlPO_4_, were 0–4.62 (Figure 3E), 0–6.75 (Figure 3F), 0–13.58 (Figure 3G), and 1.00–8.50 (Figure 3H), respectively. The *A. pittii* gp-1 presented good performance for utilizing these four types of P sources in both liquid and solid medium, with P dissolving level ranging from 7.91 to 250.77 mg/L and from 3.80 to 7.11 mg/L, respectively. These results suggested that *A. pittii* gp-1 had great potentials for multiple P sources utilization.

The details of the content of WSP and AP under soil culture conditions after 30 days’ incubation are shown in Table 3. The WSP and AP content achieved the highest value under conditions with *A. pittii* gp-1 addition in the same soil incubation group. In S + B group, S + B + T group, and S + B + T + N group, the WSP content in soils with *A. pittii* gp-1 addition were 0.42, 0.52, and 0.80 mg/g, respectively; while AP content in soils with *A. pittii* gp-1 addition were 0.51, 0.81, and 1.64 mg/g, respectively. Additionally, we found the content of WSP and AP in S + B + T + N group were significantly higher than that in S + B group and S + B + T group (*P* < 0.05) (Table 3). Most PSB with the addition of Ca_3_(PO_4_)_2_ could also significantly increase the content of WSP and AP. These results indicated that *A. pittii* gp-1 presented great utilization potential for enriching soil plant-absorbable P in practical condition.

**TABLE 3:** Evaluation of phosphate-solubilizing abilities on soil incubation.

Strains	Soil + PSB		Soil + PSB + Ca_3_(PO_4_)_2_		Soil + PSB + Ca_3_(PO_4_)_2_ + Nutrient	
	WSP (mg/L)	AP (mg/L)	WSP (mg/L)	AP (mg/L)	WSP (mg/L)	AP (mg/L)
gp-1	0.42(a)(C)	0.51(a)(C)	0.52(a)(C)	0.81(a)(B)	0.80(a)(B)	1.64(a)(A)
gp-2	0.31(ab)(C)	0.37(ab)(C)	0.40(abc)(C)	0.69(abc)(B)	0.71(ab)(B)	1.47(ab)(A)
gp-3	0.31(ab)(C)	0.40(ab)(C)	0.39(abc)(C)	0.62(bc)(B)	0.69(abc)(B)	1.44(ab)(A)
gp-4	0.26(ab)(D)	0.42(ab)(BCD)	0.39(abc)(CD)	0.60(bc)(BC)	0.61(abcde)(B)	1.41(ab)(A)
gp-6	0.29(ab)(B)	0.39(ab)(B)	0.34(c)(B)	0.56(bcd)(B)	0.67(abcd)(B)	1.24(ab)(A)
gp-7	0.19(b)(D)	0.33(ab)(CD)	0.38(abc)(CD)	0.52(cd)(BC)	0.65(abcd)(B)	1.33(ab)(A)
gp-8	0.17(b)(C)	0.30(b)(C)	0.33(c)(BC)	0.38(d)(BC)	0.58(abcde)(B)	1.04(b)(A)
gp-11	0.33(ab)(C)	0.50(a)(BC)	0.40(abc)(C)	0.74(ab)(B)	0.59(abcde)(BC)	1.35(ab)(A)
gp-12	0.26(ab)(D)	0.37(ab)(CD)	0.33(c)(CD)	0.62(bc)(B)	0.53(bcde)(BC)	1.41(ab)(A)
gp-15	0.31(ab)(D)	0.41(ab)(BCD)	0.38(abc)(CD)	0.62(bc)(B)	0.55(bcde)(BC)	1.48(ab)(A)
Ap-1	0.25(ab)(C)	0.30(b)(C)	0.33(c)(C)	0.59(bc)(B)	0.51(bcde)(B)	1.39(ab)(A)
Ap-2	0.22(b)(C)	0.33(ab)(BC)	0.37(bc)(BC)	0.53(cd)(B)	0.45(de)(BC)	1.15(ab)(A)
Ap-3	0.22(b)(D)	0.35(ab)(CD)	0.32(c)(CD)	0.57(bcd)(B)	0.47(cde)(BC)	1.14(b)(A)
Ap-4	0.27(ab)(C)	0.39(ab)(BC)	0.38(abc)(BC)	0.55(bcd)(B)	0.49(bcde)(BC)	1.16(ab)(A)
Ap-6	0.30(ab)(B)	0.36(ab)(B)	0.41(abc)(B)	0.54(cd)(B)	0.51(bcde)(B)	1.19(ab)(A)
Ap-8	0.23(b)(C)	0.30(b)(C)	0.38(abc)(BC)	0.58(bc)(B)	0.40(e)(BC)	1.13(b)(A)
Ap-9	0.33(ab)(B)	0.36(ab)(B)	0.45(abc)(B)	0.62(bc)(B)	0.52(bcde)(B)	1.14(b)(A)
Ap-10	0.33(ab)(C)	0.44(ab)(BC)	0.49(ab)(BC)	0.65(abc)(B)	0.65(abcd)(B)	1.46(ab)(A)

*The results are the mean value of two replicates. The lowercase letters in the same column represent significance (P < 0.05), the capital letters in the same row denote significant level (P < 0.05).*

### Effect of Tricalcium Phosphate and Strain gp-1 on Pb Immobilization and P-Cycling-Related Gene Abundance

**FIGURE 4:**
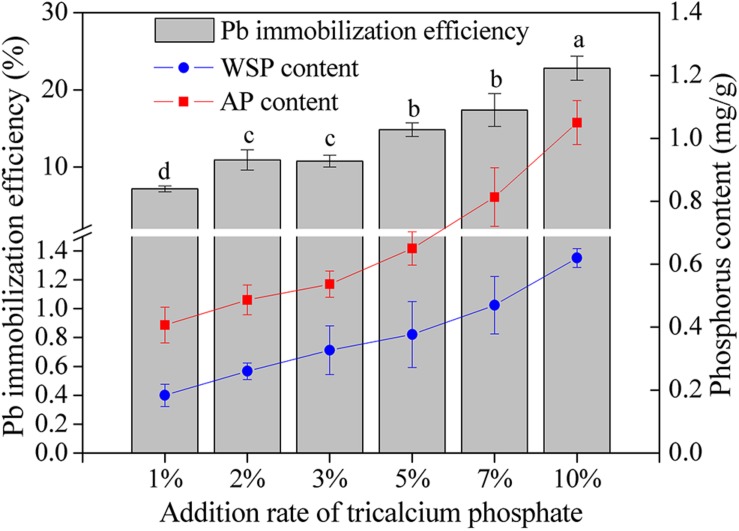
The Pb immobilization efficiency and phosphorus content in soils with different addition rate of tricalcium phosphate. The results are the mean value of three replicates, error bars represent standard error. The lowercase letters above the columns denote significant level (*P* < 0.05).

The content of WSP and AP and the Pb immobilization efficiency increased as the addition rate of tricalcium phosphate increased (Figure 4). When the addition ratio of tricalcium phosphate was 10%, the WSP and AP reached the highest value of 0.62 and 1.05 mg/L, respectively. The Pb immobilization efficiency increased from 7.16% at tricalcium phosphate addition ratio of 1 to 22.80% at tricalcium phosphate addition rate of 10%. Additionally, the Pb immobilization efficiency was significantly correlated with WSP (*r* = 0.987, *P* < 0.01) and AP (*r* = 0.990, *P* < 0.01) (Table 4).

**TABLE 4:** Pearson correlation analysis among phosphorus content, Pb immobilization efficiency and gene abundance.

Type	WSP	AP	Pb-IE^a^	*bpp*	*phoD*	*gcd*
WSP	1	0.990**	0.987**	0.932**	0.577	0.848*
AP	0.990**	1	0.990*	0.906*	0.566	0.772
Pb-IE	0.987**	0.990**	1	0.917*	0.640	0.817*
*bpp*	0.932**	0.906*	0.917*	1	0.747	0.889*
*phoD*	0.577	0.566	0.640	0.747	1	0.677
*gcd*	0.848*	0.772	0.817*	0.889*	0.677	1

^a^Pb-IE, Pb immobilization efficiency. The asterisks represent significant level (* P < 0.05; ** P < 0.01).

**FIGURE 5:**
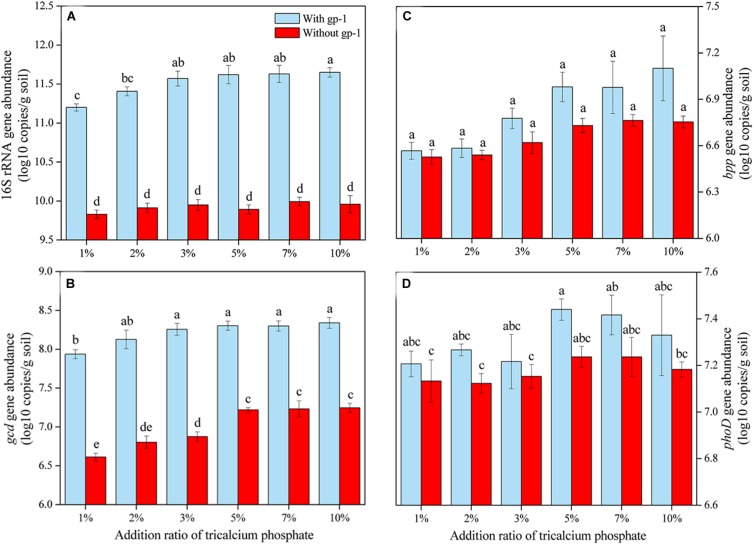
The abundances of 16S rRNA gene **(A)**, *gcd* gene **(B)**, *bpp* gene **(C)**, and *phoD* gene **(D)** in soils with different addition rate of tricalcium phosphate. The results are the mean value of three replicates, error bars represent standard error. The lowercase letters above the columns denote significant level (*P* < 0.05).

The abundances of 16S rRNA genes, *gcd*, *bpp*, and *phoD* in soils without both strain gp-1 and Ca_3_(PO_4_)_2_ addition were 6.37 × 10^9^, 4.08 × 10^6^, 3.38 × 10^6^, and 1.34 × 10^7^ copies/g, respectively. The addition of tricalcium phosphate and *A. pittii* gp-1 presented different effects on the abundances of P-cycling-related genes (Figure 5). The 16S rRNA gene abundance (6.76 × 10^9^–9.12 × 10^11^ copies/g) slightly increased as the Ca_3_(PO_4_)_2_ addition ratio increased in soils without gp-1 addition (*P* > 0.05; Figure 5A). While 16S rRNA gene abundance in soils with gp-1 addition was significantly higher than that in soils without gp-1 addition and in soils without both gp-1 and Ca_3_(PO_4_)_2_ addition (*P* < 0.05), and the 16S rRNA gene abundance increased from 1.58 × 10^11^ copies/g at Ca_3_(PO_4_)_2_ addition ratio of 1% to 4.47 × 10^11^ copies/g at Ca_3_(PO_4_)_2_ addition ratio of 10%. These results indicated that the significant increase of bacterial abundance might be due to the retained PSB *A. pittii* gp-1. Besides, *gcd* gene abundance in gp-1 added soils (ranging from 8.64 × 10^6^ copies/g to 2.19 × 10^8^ copies/g) was significantly higher than that in no gp-1 added soils (ranging from 4.11 × 10^6^ copies/g to 1.76 × 10^7^ copies/g) and in soils without both gp-1 and Ca_3_(PO_4_)_2_ addition (*P* < 0.05; Figure 5B). At the same ratio of tricalcium phosphate addition, we found that *bpp* gene abundance in soils with gp-1 addition (3.69 × 10^6^–1.26 × 10^7^ copies/g) was higher than that in soils without gp-1 addition (3.36 × 10^6^–5.80 × 10^6^ copies/g) and in soils without both gp-1 and Ca_3_(PO_4_)_2_ addition (*P* > 0.05; Figure 5C). However, the *phoD* gene abundance in with gp-1 added soils (1.61 × 10^7^–2.75 × 10^7^ copies/g) was slightly higher than that in without gp-1 added soils (1.33 × 10^7^–1.72 × 10^7^ copies/g) and in without both gp-1 and Ca_3_(PO_4_)_2_ added soils (*P* > 0.05; Figure 5D). The Pb immobilization efficiency was significantly correlated with *bpp* (*r* = 0.917; *P* < 0.05) and *gcd* (*r* = 0.817; *P* < 0.05) gene abundance (Table 4). These results indicated that P solubilization was responsible for the immobilization of Pb. In addition, *gcd* could be amplified from *A. pittii* gp-1 using the primers described above, while *bpp* and *phoD* did not. These results implied that the addition of *A. pittii* gp-1 could increase the abundances of organic P-cycling-related genes.

### Changes in *ppk* and *pqq* Gene Expression Level

**FIGURE 6:**
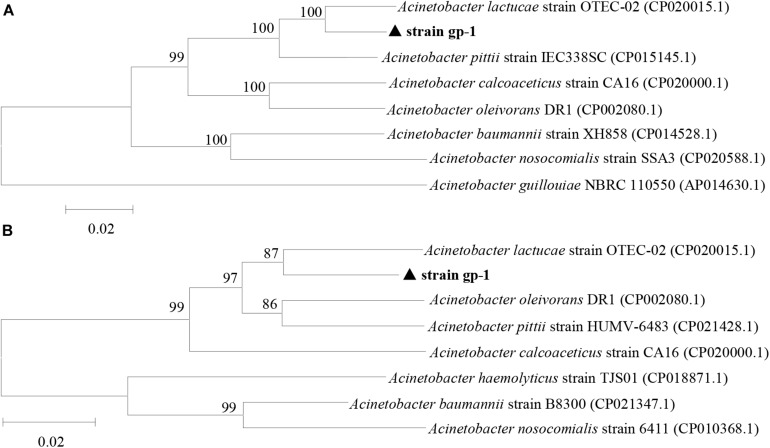
Neighbor-joining phylogenetic tree of *ppk* gene **(A)** and *pqq* gene **(B)** based on sequence similarity. The numbers at the nodes indicate the levels of bootstrap support based on data for 1000 replicates. The scale bar represents 2% sequence divergence.

**FIGURE 7:**
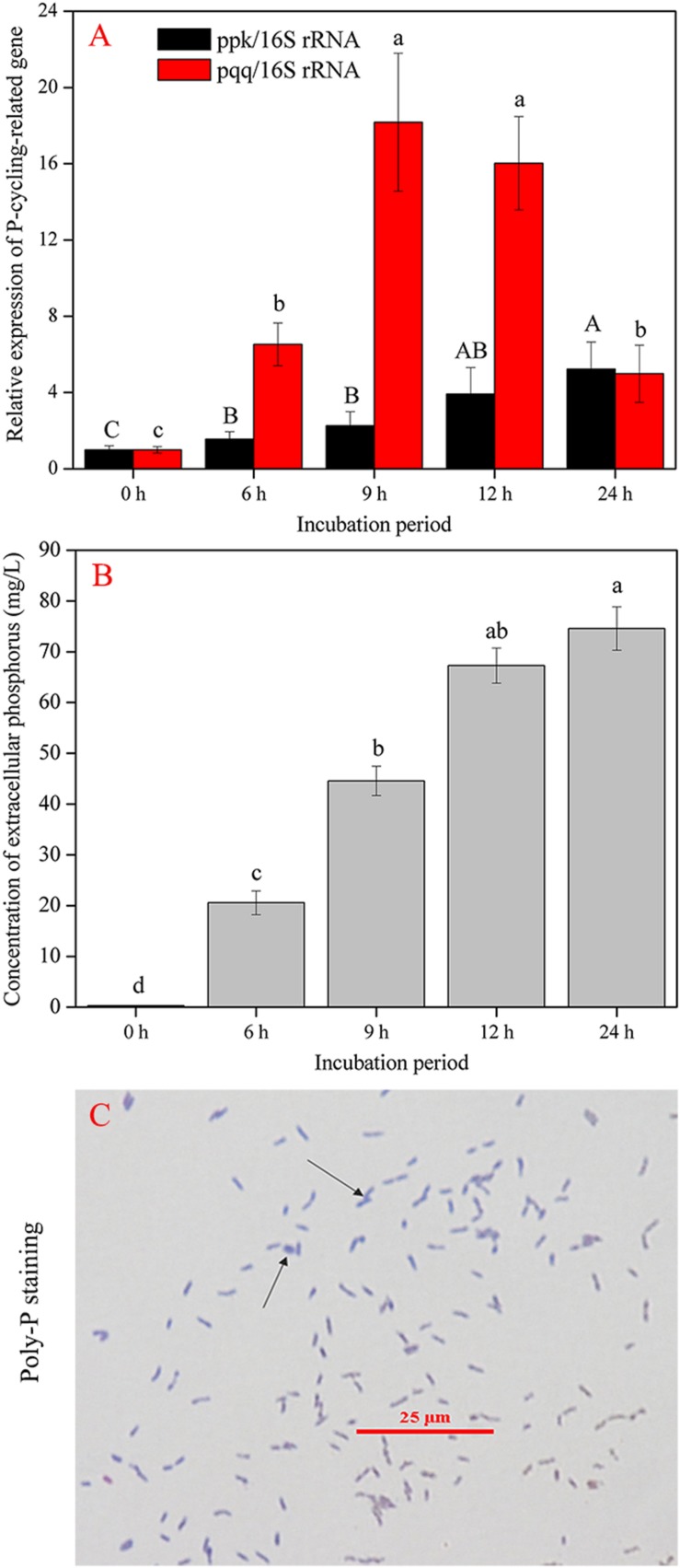
Gene abundance and phosphorus determination during 24 h. **(A)** shows the shift in P-cycling-related gene abundance. **(B)** reflects the concentration of extracellular phosphorus. **(C)** shows the granular staining. The results are the mean value of three replicates, error bars represent standard error. The lowercase letters above the columns denote significant level (*P* < 0.05).

The complete sequences of *ppk* and *pqq* genes in *A. pittii* gp-1 presented the highest similarities with complete sequences of *ppk* and *pqq* genes in *Acinetobacter lactucae* strain OTEC-02 (accession number: CP020015.1) (Figure 6), with corresponding similarities of 96.01% (Figure 6A) and 94.98% (Figure 6B), respectively. The expression levels of P-cycling-related genes and concentration of free phosphorus presented dynamic change during strain gp-1 incubation (Figure 7). The relative expression level of *pqq* gene significantly (*P* < 0.05) increased 18.18 times at the 6th h, and then declined to 4.99 times at the 24th h (Figure 7A). While the relative expression level of *ppk* gene dramatically (*P* < 0.05) increased, and achieved the highest value of 5.23 times at the 24th h. Similarly, the concentration of extracellular phosphorus remarkably increased from 0.29 mg/L at the initial period to 74.58 mg/L at the 24th h (*P* < 0.05; Figure 7B). The extracellular phosphorus concentration was positively correlated with the relative expression levels of *ppk* (*r* = 0.951; *P* < 0.05) and *pqq* (*r* = 0.495; *P* > 0.05). In addition, granule staining presented positive reaction (Figure 7C), suggesting Poly-P existed in *A. pittii* gp-1 cells. These results implied that *ppk* and *pqq* genes were closely correlated with phosphorus cycling, and the process of extracellular inorganic insoluble phosphorus solubilization was coupled with the process of polyphosphate synthesis.

## Discussion

### Assessment of Diversity and Phosphorus Solubilizing Capacity of PSB

In this study, we found big shifts in bacterial community composition during four acclimation stages. Some PSB have been identified belonging to *Proteobacteria*, *Bacteroidetes*, *Firmicutes*, and *Deinococcus–Thermus* (Vyas and Gulati, 2009; Shrivastava et al., 2010; Hanif et al., 2015; Li et al., 2017), which were also the major bacterial phylum in our study. Some genera, such as *Sphingobacterium* and *Cellvibrio*, have not been reported as PSB, however, they were the major genus in four acclimation stages. This might be due to they are potential PSB could not be cultured at present, or they are closely related with PSB.

After four rounds of acclimation, we gained 18 PSB belonging to eight genera including *Acinetobacter*, *Arthrobacter*, *Bacillus*, *Cupriavidus*, *Massilia, Ochrobactrum*, *Pseudomonas*, and *Stenotrophomonas*. Previous literatures have reported that some PSB belong to these genera (Yu et al., 2011; Imran et al., 2014; Liu et al., 2014; Silva et al., 2017), but most studies have not reported PSB possessing multiple P sources utilizing abilities. For instance, strain gp-1 identified as *Acinetobacter* genus in our study presented good performance for solubilizing Ca_3_(PO_4_)_2_, FePO_4_, and AlPO_4_, and for digesting phytate, with corresponding P solubilizing levels were 250.77, 46.10, 81.99, and 7.91 mg/L PO_4_^3–^-P, respectively. The P solubilizing capacity of *A. pittii* gp-1 is higher than that of other reported bacteria, such as *Acinetobacter baylyi* W16 for Ca_3_(PO_4_)_2_ (91 mg/L) (Yu et al., 2011), *Enterobacter* sp. C for Ca_3_(PO_4_)_2_ (100 mg/L) (Melo et al., 2018), *Bacillus* sp. AB066338 for Ca_3_(PO_4_)_2_ (137 mg/L) (Liu et al., 2014), *Burkholderia seminalis*. PSB7 for Ca_3_(PO_4_)_2_ (145 mg/L) (Panhwar et al., 2014), *Pantoea dispersa* Cav.cy3 for Ca_3_(PO_4_)_2_, FePO_4_, and AlPO_4_ (<50 mg/L) (Chen et al., 2014), *Pseudomonas* sp. for phytate (0.40 mg/L) (Maougal et al., 2014), and *Paenibacillus elgii* B56 for AlPO4 (16 mg/L) and *Bacillus megaterium* B119 for phytate (4.9 mg/g) (Oliveira et al., 2009). Some PSB present higher P solubilizing ability than that of *A. pittii* gp-1, such as *Pseudomonas trivialis* BIHB 745 for Ca_3_(PO_4_)_2_ (827 mg/L) (Vyas and Gulati, 2009), *Serratia marcescens* RP8 for Ca_3_(PO_4_)_2_ (974 mg/L) (Misra et al., 2012), *Acinetobacter* sp. ASL12 for Ca_3_(PO_4_)_2_ (717 mg/L) (Liu et al., 2014), *Bacillus cereus* SPC09 for phytate (579.01 mg/L) (Zhang et al., 2015), *Saccharomyces cerevisiae* CICIMY008 for phytate (93 mg/L) (Chen et al., 2016), *Burkholderia cepacia* B116 for phytate (52.7 mg/L) (Oliveira et al., 2009). However, the multiple P sources utilizing ability promises gp-1 itself easily get used to environment, and therefore exhibits great potential for phosphate chemical industry and agro-ecosystems. Additionally, the stepwise acclimation by using Ca_3_(PO_4_)_2_, phytate, FePO_4_, and AlPO_4_ provides a useful approach to obtain PSB possessing multiple P sources utilizing capacity.

### Relationship Between Pb Immobilization and P Solubilization

Many studies have reported that PSB contribute to the immobilization of heavy metal. For instance, PSB *Pantoea* sp. CS2-B1 and *Enterobacter cloacae* SM1-B1 can immobilize Pb (Park et al., 2011); *Enterobacter* sp. could enhance Pb immobilization (Chen et al., 2019); *Serratia marcescens* OPDB3-6-1 shows good capacity for the immobilization of Pb, Cd, and Cu (Zhu et al., 2019); *Pseudomonas* sp. strain PG-12 exhibits good performance for Pb immobilization (Manzoor et al., 2019); *Leclercia adecarboxylata* B3 and *Pseudomonas putida* F2-1 can turn soluble Pb(II) into insoluble form (Teng et al., 2019). The promotion mechanism of heavy metal immobilization by PSB has been acknowledged, namely PSB produce soluble phosphorus and metabolite, and then these products could bind with heavy metal ions and turn into insoluble form (Park et al., 2011; Yuan et al., 2017; Teng et al., 2019; Zhu et al., 2019). The Pb immobilization efficiency was significantly correlated with soil available P and WSP in this study, which is similar with the findings that Pb immobilized amount and available phosphorus present significant correlation (Park et al., 2011; Yuan et al., 2017). Additionally, some studies have reported that the addition of inorganic phosphorus can increase the abundance of P-cycling-related gene (Silva et al., 2017; Hu et al., 2018; Wei et al., 2019), and alter bacterial community composition (Lagos et al., 2016; Luo et al., 2017; Wei et al., 2019). However, the effect of PSB addition on indigenous organic P-cycling-related gene abundance is rarely reported. To our knowledge, we first report that the addition of *A. pittii* gp-1 can significantly increase the abundance of inorganic P-cycling-related *gcd*-harboring bacteria via direct input of *A. pittii* gp-1, and slightly increase the abundances of organic P-cycling-related *bpp*-harboring and *phoD*-harboring bacterial communities via changing indigenous bacterial community. Additionally, limited study has revealed the relationship between Pb immobilization and P-cycling-related gene abundance in soils with PSB addition. The Pb immobilization efficiency was significantly positively correlated with *gcd*-harboring bacterial abundance and *bpp*-harboring bacterial abundance, suggesting *gcd*-harboring bacteria and *bpp*-harboring bacteria might be responsible for Pb immobilization. This finding might be due to on the one hand *A. pittii* gp-1 can survive from harsh condition; on the other hand, *A. pittii* gp-1 can dissolve inorganic phosphorus for enriching the soil available phosphorus, therefore causing changes in bacterial community composition and abundances of P-cycling-related genes. Previous literatures have reported that bacteria in *Acinetobacter* genus harbor heavy metal resistance gene Saffarian et al., 2017; Zhou et al., 2019), and phytase encoded by *bpp*-harboring bacteria presents high resistance to heavy metal damage (Yung et al., 2014).

### Deciphering Phosphorus Track During Inorganic Phosphorus Solubilizing

To explain the process of phosphorus metabolism of *A. pittii* gp-1, we quantified the expression of polyphosphate kinase gene (*ppk*) and pyrroloquinoline quinone (*pqq*). Our results revealed that the transformation of insoluble tricalcium phosphate into soluble phosphorus involved in a strong expression of *pqq* gene. This finding is in line with previous reports that the expression of *pqq* gene is closely correlated with the solubilization of inorganic phosphorus (Ogut et al., 2010; Wagh et al., 2014; Oteino et al., 2015). We also observed an increasing expression level of *ppk* gene, which was closely correlated with the content of extracellular P. In addition, we found the existence of polyphosphate by using granular staining. To our knowledge, this is the first report that the solubilization of inorganic insoluble phosphorus is coupled with the synthesis of polyphosphate. It has been reported that polyphosphate regarded as high-energy compound could be hydrolyzed when phosphorus accumulating bacteria undergo undernourished conditions (Wan et al., 2017; Li and Dittrich, 2019; Zhong et al., 2019). Previous literatures have reported that some bacteria belonging to *Acinetobacter* genus harbor the ability to synthesize polyphosphate and could be regarded as phosphorus accumulating bacteria (Keating et al., 2016; Han et al., 2018). Therefore, we can conclude that one part of soluble phosphorus can be used for the formation of bacterial substance (e.g., DNA and RNA), and another part of soluble phosphorus can be applied for the synthesis of polyphosphate during inorganic phosphorus solubilization. However, the process of polyphosphate synthesis should be inhibited or blocked when use PSB in agro-ecosystems, since polyphosphate is not plant-absorbable phosphorus.

## Conclusion

In this study, we found big changes in diversity and composition of bacterial community during four acclimation periods. *Cellvibrio* was dominant genus in the first and second rounds of acclimation, while *Sphingobacterium* was the dominant genus in the third and fourth rounds of acclimation. A total of 18 PSB belonging to *Acinetobacter*, *Arthrobacter*, *Bacillus*, *Cupriavidus*, *Massilia, Ochrobactrum*, *Pseudomonas*, and *Stenotrophomonas* presented multiple phosphorus sources utilizing capabilities. The isolate *A. pittii* gp-1 exhibited good performance for utilizing Ca_3_(PO_4_)_2_, phytate, FePO_4_, and AlPO_4_ in both solid and liquid medium. Additionally, strain gp-1 could significantly increase the content of soil available phosphorus and presented good capacity in immobilizing Pb. Simultaneously, the addition of *A. pittii* gp-1 could increase the abundance of P-cycling-related genes including *gcd*, *bpp*, and *phoD*. To our knowledge, we first report that the solubilization of tricalcium phosphate by phosphorus solubilizing bacterium belonging to *Acinetobacter* is coupled with the synthesis of polyphosphate.

Therefore, soil-derived *A. pittii* gp-1 possessing multiple P sources utilizing ability and Pb immobilization capacity exhibits great potentials in agro-ecosystems.

## Data Availability Statement

The datasets generated for this study can be found in NCBI BioProject PRJNA527148, accession numbers SRR8731888–SRR8731891.

## Author Contributions

WW designed the whole experiment. WW, YQ, HW, WZ, HH, JT, and YW conducted all the experiments. WW analyzed the data and wrote the manuscript. DH revised the manuscript.

## Conflict of Interest

The authors declare that the research was conducted in the absence of any commercial or financial relationships that could be construed as a potential conflict of interest.

## Footnotes

1 http://www.ncbi.nlm.nih.gov

2 http://blast.ncbi.nlm.nih.gov/Blast.cgi
